# A Case of Metastatic Abscess, Following Compound Fracture of the Thigh, Rupture of the Spleen

**Published:** 1853-02

**Authors:** 


### ART. III.—*A Case of Metastatic Abscess, following Compound Fracture of the Thigh, Rupture of the Spleen.*

This patient was a boy of fourteen. Previous to his tenth year he had been generally healthy, but for the last four or five years he had grown pallid and slender, though without exhibiting any other very definite morbid symptom. On the afternoon of July 30th, as he was playing about the decks of a vessel, he fell through a scuttle, a distance of something over ten feet, striking on a pile of ring-bolts and other pieces of iron. He was stunned for a few minutes, but soon recovered. He made no complaint of pain except in his "stomach." He was at first somewhat collapsed, but grew warm soon after being put to bed.

When first seen by a medical man, about an hour and a half after the accident, he was lying quiet in bed, making no complaint; but when interrogated said he had pain in the left thigh and left hypogastrium. Countenance pale. Skin moderately warm. Pulse 60, of good force.

The left femur was found to be fractured a little below its middle; and one and a half to two inches shortened. The lower fragment was drawn inward, and, together with the leg and foot, considerably *inverted*, so that the foot rested on its inner edge. The upper fragment was directed outward. There was a wound on the outside of the thigh, one inch in length. The finger introduced into the wound, passed upward and inward for about one and a half inches, and came in contact with the lower extremity of the upper fragment of the femur, which was stripped bare of periosteum for one inch above the fracture. The lower fragment could not be felt. The wound was closed by adhesive plaster, the thigh done up in a many-tailed bandage, and and the patient placed on a double-inclined plane.

He passed a tolerably quiet night. Next morning he was somewhat restless. The tongue and skin were well. Pulse 100, of good character. He had little or no pain in the thigh, but complained of tenderness over nearly the whole of the left chest. There was no bruise, however, or other injury discoverable at this part. The tumefaction of the thigh was moderate.

For two or three days succeeding he was much the same; quiet and comfortable, with some little heat of skin, and a pulse ranging from 92 to 104. His bowels were open spontaneously, and he had some appetite.

On the morning of August 3d, the fifth day of the injury, he had a well marked chill. It came on suddenly, without known cause, and continued for about ten minutes. It went off, however, after the use of a slight stimulant, and he passed the day as usual. That night he was delirious, according to the report of his nurse. Next morning (4th) he had a return of chilliness, but no distinct rigor. His countenance was then pale. Pulse 116. Manner rather flighty, but no delirium. Very talkative. Tongue natural. No appetite. A little pain in the fractured thigh.

The wound had not united at all, but otherwise there was no morbid appearance about it. The thigh was straight, and of good length and shape. The tumefaction was very moderate. He had a slight laxative of sulphate of magnesia.

Next morning (5th) he was more delirious, and the delirium had almost precisely the character of *mania a potu*. He was restless, talkative, agitated. Countenance pale. Some irregular, semi-involuntary movements of hands and fingers, approaching to tremor. He appeared sometimes to see objects in the room which had no existence, as "mummies," &c. Pulse 104. Skin and tongue as the day previous. During this day he had the elixir of opium twice, in doses of ten drops, but it did not appear to quiet him in the least.

On the 6th August, (eighth day of the injury,) a swelling was first observed on the back of the right hand, which had increased by the next day (7th.) The general symptoms were also more unfavorable. The delirium continued. The countenance was more haggard than before. Teeth and lips dry, and covered with brown sordes. Tongue dryish, with a thin yellowish coat. Thirst constant. He had slept much at intervals since the preceding evening, but was easily roused. While awake he was anxious, querulous, restless. He cried out often, as if in pain or fear, but could not indicate distinctly the seat of his distress. He had taken five doses of elixir of opium since the morning of the fifth. The wound discharged no pus, but only a thin reddish fluid. The swelling on the back of the hand was moderately firm, situated chiefly over the back part of the carpus and meta-carpus. The integuments were œdematous, of a diffused dull red color, which vanished under the pressure of the finger and returned again immediately. There was much tenderness of the hand on firm pressure, but the patient did not complain of pain in this part except during examination. There was no fluctuation.

There was also a decided fullness, without any discoloration of the skin over the posterior part of the left shoulder joint, and the adjoining parts of the scapula. The patient complained bitterly when the left arm was moved, and talked much, in his delirium, of a "dog" having "bitten him there."

The elixir of opium was suspended, and he was ordered wine and water, $\overline{aa}$ ℥ss. every two hours.

He took the wine without difficulty, and the next day, as he had not lost ground at all, the dose of the stimulus was reduced to ℥ss every four hours; and he continued to take it in this quantity. The swelling of the right hand gradually increased, and extended up the fore-arm to the elbow, the redness of the skin continuing of the same character as at first, and confined altogether to the back of the hand. The swelling of the left shoulder increased but little, and though a slight redness appeared over the upper and outer part of the joint, it was never well-marked or extensive.

The delirium gradually altered in character and became more quiet and heavy, resembling that of typhoid fever. The skin was a little hot and rather moist, but there was at no time excessive heat or profuse perspiration. Pulse 112 to 120, moderately strong and full.

On the morning of the 9th, his bowels having been opened three times on the preceding day, without medicine, he had a fluid, light-colored, involuntary discharge; and soon after, having been left for a few moments by the attendant in charge of him, he fell from the bed on to the floor, a distance of about three feet. His fall was light, and he remained after it lying quietly on the floor, without making any outcry or other expression of pain. The thigh did not appear to be displaced, nor were there any bruises or other marks of injury consequent on the fall.

At this time his countenance was pallid and haggard as before; the skin moderately warm and moist; the tongue and lips dry. Pulse 112. He was sufficiently intelligent to put out his tongue when desired to do so, but talked irrationally and with an indistinct articulation. The pupils were equal, of natural size and sensible to light.

Copious fluid, involuntary, discharges from the bowels continued through the 9th and 10th. On the 11th there was no remarkable change, except that the countenance was more sunken. Skin warm. Pulse 136, and feebler than before. He took some beef tea, apparently with relish. He seemed to be free from pain. He got the following prescription:

℞ Mist. Cretæ ℥ss.
Elixir Opii, gtt. x.
M.

On the evening of the 11th he appeared weaker. Pulse 140, and the skin inclined to be cool. He screamed out, from time to time, and talked unintelligibly. The articulation was very indistinct. He died on the morning of the 12th, without convulsion, stertor, or any other remarkable symptom.

He had no cough at any time during his illness. On the 10th his respiration was recorded as 48 per minute, but it was not at all laborious, and resembled simply the rapid respiration of constitutional irritation. He occasionally complained to those about him of pain in the abdomen, but this was not a prominent symptom, and never sufficient to attract much attention after the first two days of the accident.

*Autopsy* four hours after death. Cadaveric rigidity tolerably well established. Countenance natural. No remarkable discoloration of skin.

HEAD.

The longitudinal sinus filled with a dark coagulum. A drachm or two of reddish serum at the base of the brain. Brain and membranes everywhere quite healthy. No softening, vascularity, discoloration, or other morbid appearance anywhere.

### CHEST.

*Heart, &c.* Pericardium natural, containing the normal amount of clear serum. Right cavities of the heart considerably distended with dark fluid and coagulated blood, and fibrinous clots; the latter very firm in consistency. Venæ cavæ and pulmonary artery each contained dark fluid blood and some dark coagula. The left ventricle was firm, prominent, nearly empty. The left auricle contained a little dark-red and fibrinous clot. Endocardium and valves all healthy. Aorta, carotid arteries and jugular veins ditto. Weight of heart 5½ ounces.

*Lungs, &c.* Anterior surface of both lungs of a light pinkish color. Both pleuræ healthy. No effusion. Upper and middle lobes of right lung very full of air. These parts did not collapse so much as usual after opening the chest, and were throughout of a light pink color. Pulmonary vesicles larger than usual.

*Pneumonia.* The lower lobe, excepting a very small portion of its upper and anterior part, was universally of a dark purple color, heavy and solid in consistency, and having a finely lobulated feel, like lung thickly filled with tubercle. The posterior surface showed numerous dark-red circular spots of ecchymosis, apparently in the substance of the pleura, or immediately beneath it. This lobe did not crepitate; and, when the remaining portions of the lung were distended by inflation, admitted the air very imperfectly, and retained nearly its original size and shape. Its section presented a dark-purple granulated surface, without pus, or much fluid exudation of any kind. Its texture was considerably softened. There was no alteration of the bronchi in the inflamed portion of the lung. The line of demarkation between the inflamed and healthy parts was very distinct. The left lung was for the most part quite healthy, only a small portion of the posterior edge of the lower lobe was inflamed, like the lower lobe of the right. The left lung weighed five ounces; the right eleven ounces.

There was a small flake of yellow lymph between the adjacent edges of the lower and middle lobes of the right lung; but this was the only alteration of the pleura discoverable anywhere.

### ABDOMEN.

*Blood in Peritoneum.* The peritoneal cavity contained about an ounce and a half of dark fluid blood, most of which was collected in the lower part of the pelvis, and the remainder smeared over the surface of the liver and intestines. The peritoneum itself was not altered. No lymph or unnatural adhesions anywhere. The exterior of the intestines was stained in some places of a dark-brown, almost black color. In the left iliac region, the mesocolon had some dark ecchymosis between its layers of peritoneum.

*Alimentary Canal.* The œsophagus was natural. The stomach empty. The gastric mucous membrane was not remarkable except for some cadaveric softening, with brownish discoloration of the blood vessels in the cardiac extremity. It was very much corrugated. The small intestine contained a little orange-yellow pasty matter. Peyer's patches moderately well-developed. Solitary glandulæ, especially near the termination of ileum, very much so. Large intestine natural. Cæcal glandulæ much developed. The liver was, in every respect, natural. It weighed two pounds, eight and a half ounces. The gall bladder contained about an ounce of dark brown bile.

*Fracture of Spleen.* The spleen was of the natural size and adhesions. It had evidently been fractured, at no very distant period, in a transverse direction. There was a line running across its surface, when the peritoneal covering and the substance of the spleen itself had been torn apart, and the interval filled up with lymph. The torn edges of the peritoneum were tolerably straight and regular, and were separated from each other by an interval of half an inch. The lymph that filled up the fissure, projected a little above the surrounding surface of the organ, and overlapped the torn edge of the peritoneum for a line or two. The fracture extended completely across the body of the spleen. The "filling up" of lymph was, at one end, yellow and quite hard, but, for the most part, was of a moderately firm consistency, of a dull reddish color, and of a somewhat granulated texture. There were two or three small cavities in its substance, containing old, dark-colored fluid blood. The remaining portion of the spleen was of healthy appearance and consistency. Its peritoneal covering was generally smooth and shining.

The pancreas was natural. Kidneys ditto. The bladder contained a few ounces of clear urine. Its mucous membrane and other coats were healthy.

The abdominal aorta, iliac arteries and veins, and the abdominal vena cava were all healthy.

### LEFT SHOULDER.

There was a nearly uniform, smooth tumefaction of the left shoulder, as during life, with little or no discoloration of the skin. It was most prominent on its posterior aspect, just above the axilla. On cutting through the skin and subcutaneous cellular and fatty tissue at this part, an abscess was opened, from which a quantity of thick, yellow, normal-looking pus flowed out. From this opening, situated just at the posterior edge of the deltoid muscle, the abscess extended backward on to the dorsum of the scapula, underneath the fascia covering the infra-spinatus muscle. The walls of the abscess were here rather ill-defined, — not at all indurated, — and appeared to consist of fibrous tissue, of which the connecting cellular membrane had been destroyed, and replaced by pus, or soft yellow lymph. The infra-spinatus, and other muscles in the immediate neighborhood, were quite natural in color and consistency. From this part the abscess extended forward, and communicated with the shoulder-joint, the capsule of which was open on its outer and posterior aspect, and which contained pus, of the same quality as that elsewhere. From the cavity of the joint the abscess extended in various directions; backward, among the muscles filling the supra-spinous fossa of the scapula; inward, beneath and in front of the clavicle, quite half way to the sterno-clavicular articulation; two or three inches downward upon the side of the chest, beneath the great pectoral muscle; and a little over half way down the posterior and inner part of the humerus. The walls of the abscess were everywhere nearly similar in appearance, and the muscles in its neighborhood were everywhere red and firm. The whole amount of pus was estimated at an ounce and a half. No bone was exposed anywhere. The articulating surfaces of the humerus and glenoid cavity were absolutely healthy in appearance. The cartilages smooth, hard, yellowish-white in color, and of natural adhesion to the bone.

### RIGHT WRIST.

There was much œdema of the right hand and fore-arm, as during life. On cutting through the integuments and fascia in front of the wrist there was found to be a collection of thick, yellow pus among the deep-seated muscles and tendons of the fore-arm and hand. The tendons themselves were white and hard, with only a very little vascularity here and there; hardly enough to attract notice. The general appearance of the pus, cavity, and walls of the abscess were the same here as in the left shoulder. The abscess extended up the fore-arm, among and beneath the deep muscles, nearly to the elbow-joint; down the palmar and dorsal aspects of the hand, nearly to the extremity of the metacarpus; and, by several openings in the anterior ligament of the wrist, communicated with the cavity of the joint; and, finally, also with the cavity of the articulation between the first and second rows of carpal bones. The articulating surfaces were natural, except for a small spot on the lower end of the radius, where the cartilage appeared thinned, so that the red color of the bony surface showed through it.

The brachial, radial, and ulnar arteries were natural. The basilic, cephalic, and brachial veins were also natural, externally and internally.

#### LEFT THIGH.

There was hardly any tumefaction of the thigh, or infiltration of the tissues The fractured extremities of the femur were shot past each other about an inch. The lower two inches of the upper fragment was destitute of periosteum, white, and hard; and, on being struck with the back of the knife, returned a sharp clicking sound, like ordinary dead bone. The end of the lower fragment was in the same condition for the extent of one inch. The cavity of the wound was not very extensive. It contained no pus, but only a little thin, dirty, greyish-yellow fluid. Its internal surface was covered with yellowish and ashy-colored lymph. The muscles and other soft parts of the thigh were everywhere of a healthy appearance.

The femoral artery and vein were natural.

